# Peters' Anomaly – Anaesthetic Management

**Published:** 2009-08

**Authors:** Senthilkumar M, Darlong V, Jyotsna Punj, Ravinder Pandey

**Affiliations:** 1 Senior Resident, Department of Anaesthesiology & Intensive Care Unit, All India Institute of Medical Sciences, New Delhi; 2,3,4 Assistant Professor, Department of Anaesthesiology & Intensive Care Unit, All India Institute of Medical Sciences, New Delhi

**Keywords:** Peters' anomaly, Systemic malformation, Anaesthetic management

## Abstract

**Summary:**

Peters' anomaly occurs as an isolated ocular abnormality, in association with other systemic abnormality or one component of a number of well-defined syndromes. We review our experience of anaesthetic management and systemic association of peters' anomaly. To the best of our knowledge there are no reports in the literature of Peters' anomaly with relevant to anaesthesia.

## Introduction

Peters' anomaly was first described in 1906 by a German Ophthalmologist, Dr. Alfred Peters. It is characterized by a central corneal opacity due to defect in the corneal endothelium, Descemet's membrane, and posterior stroma. Systemic associations with Peters' anomaly include trisomy 13-15, partial deletion of chromosome arm 11q, and Norrie disease^1^. Major ophthalmologic complications in patients with Peters' anomaly include glaucoma and deprivation amblyopia. Despite aggressive surgical and post operative care, almost 50% of patient lose all light perception, mainly because of glaucoma.

## Case Series

Nine patients with Peters' anomaly, admitted for various eye procedures in our ophthalmic institute. Diagnosis was made by the ophthalmologist and paediatrician. Patient's characteristics are described in Table 1. All children underwent preoperative examinations and major congenital malformations were recorded. Investigations were done as per the requirement. Children age less than three years and more than 6 months were premedicated with promethazine syrup 2 mg.kg^-1^. Anaesthetic technique was planned according to their clinical involvement.

**Table 1:** Patient's data

Patient	Age(Month)	Ocular problem	Surgical procedure	Airway examination	Cardiac anomaly	Other anomaly
1	6	Corneal opacity	EUA	Micrognathia	PDA	-
2	9	Corneal opacity	EUA	Micrognathia	-	-
3	3	Cataract	EUA	-	-	-
4	3	Cataract & glaucoma	EUA	Micrognathia	-	-
5	11	Corneal opacity	EUA	-	ASD	-
6	13	Glaucoma	Trabeculectomy	-	PS	Microcephaly
7	6	Glaucoma	Trabeculectomy	Cleft lip/palate	-	-
8	1½	Glaucoma	Surgical iridectomy	Small moth opening, Micrognathia	VSD	
				Anterior larynx	-	
9	6	Corneal opactiy	Keratoplasty	-	-	-

*EUA, examination under anaesthesia; PDA, patent ductus arteriosus; VSD, ventricular septal defect; PS, pulmonary stenosis; ASD, atrial septal defect;

Five patients admitted for examination under anaesthesia (EUA). In the operating table, ECG, non invasive blood pressure and oxygen saturation were established (Datex Ohmeda). General anaesthesia was induced with O_2_:N_2_O and sevoflurane 5 - 8%. Intravenous (IV) access was secured after induction. Anaesthesia was maintained with using O_2_:N_2_O: isoflurane mixture. In all children spontaneous respiration was maintained with a face mask attached to T – piece. Laryngeal mask airway (LMA) was inserted in two patients because of difficulty in holding mask due to mandibular hypoplasia. The procedures were completed without any complications.

Three patients were operated for optical iridectomy and one for keratoplasty. Anaesthesia was induced with inhalational agent, using O_2_:N_2_O and sevoflurane 5 - 8%. IV line was secured after induction and fentanyl was given 1-2 mcg.kg^-1^ for analgesia. LMA was inserted when an adequate depth of anaesthesia was achieved (MAC-LMA insertion for sevofluranre 2.0-2.5%). Ana esthesiawas maintained with O_2_:N_2_O: isoflurane mixture and pressure-controlled ventilation was used intraoperatively by adjusting the pressure to maintain normocarbia. Muscle relaxation was achieved using atracurium (0.1mg. kg^-1^) and rectal paracetamol (40mg.kg^-1^) was inserted in all the patients for postoperative analgesia. We encountered difficulty in intubating the trachea of 1½ month old baby who was operated for iridectomy. Tracheal intubation was planned but couldn't be intubated in the fast attempt due to mandibular hypoplasia and anterior larynx. Finally intubation was done after fifth attempt with stylet and BURP maneuver.

Intraoperatively ECG, non-invasive blood pressure, end-tidal carbon dioxide and oxygen saturation were monitored. At the end of surgery, neuromuscular blockade was reversed with neostigmine (50-70mcg.kg^-1^) and glycopynolate (10mcg.kg^-1^). Extubation was performed with the child fully awake. All patients were shifted to post anaesthesia recovery room for monitoring and oxygen was administered by face mask till they were fit to be discharged or shifted to ward.

## Discussion

Anaesthetic management of Peters' anomaly varied depending on the clinical manifestations and associated other anomalies. Anaesthetic consideration includes possibility of difficult airway, presence of congenital heart disease and other systemic anomalies. Systemic associations are described in the Table 2. Midline body structures seem to be involved. All associated systemic anomalies appeared to arise from maldevelopment of the neural crest cells^2^.

**Table 2:** Systemic features of Peters' anomaly

	Peters' Anomaly
• Genetic	Sporadic, Autosomal recessive & dominant
• Ocular	Sclerocornea, Cataract, Glaucoma, Microphthalmia, coloboma
• Airway	Micrognathia, Cleftlip/palate, Anterior larynx, Depressed nasal bridge, Laryngomalacia, Subgloticstenosis, Macroglossia
• Cental nervous system	Microcephaly, Hydrocephalus, Cerebral atrophy, Seizure Spina bifida
• Cardivascular system	VSD, PDA, ASD, Tetraologyof fallot, duplicated / right aortic arch, Dextrocardia
• Respiratory system	Pectus excavatum, Pulmonary hypoplasia, Kyphoscoliosis
• Renal system	Pyelonephritis, Hydronephrosis
• Musculoskeletal system	Short limb dwarfism, Hypo/Hyper joint mobility, Hypotonia
• Endocrine system	Pituitary dysfunction
• Gastrointestinal system	Intestinal malrotation
• Other	Hearing loss

Most common heart disease is acyanotic heart disease with leftto right shunt. In our case series, four patients had acyanotic heart disease with left to right shunt (VSD, PDA, ASD). Inhalational induction was done in all children using sevoflurane as sevoflurane has been shown to preserve myocardial function better in patients with congenital heart disease (CHD)as compared with halothane^3^. In our case series there were no significant hemodynamic changes after induction.

In Peters' anomaly airway management may be difficult in patients with significant dysmorphic facial features, especially with micrognathia and cleft lip/palate. The anterior mandibular space is reduced due to micrognathia, thus makungtracheal intubation more difficult in Peters' anomaly. In our case series, five patients had anticipated difficult airway problems (micrognathia, cleftlip/palate, smallmouth opening, anterior larynx). We used LMA in these patients without any difficulty. The otheradded advantages of using LMA in ophthalmic procedure are: (i) intraocular pressure and coughing are lower with the LMA than the tracheal tube (TT) (ii) Anaesthetic depth requirements are lower for the LMA than the TT^4^. Fibreoptic intubation should be considered in patient with limited mouth opening and cervical vertebral anomaly. Although not a significant feature of this syndrome, patients with hyperor hypo mobility joint disease may require careful positioning and padding.

Mental retardation is common in majority of these patients and seizures have been less reported. In our case series none of the children had mental retardation and seizures. Mental retardation may limit patient cooperation, therefore sedative and/or anxiolytic premeditation maybe helpful. In 1984, van Schooneveld et al first proposed the term Peters'-plus syndrome, comprising Peters anomaly, face anomalies, clefting, short limb dwarfism, and retarted development^5^. In our case series, some patients presented with some of these findings. However, no cases completely fulfilled the criteria for this syndrome. One important differential diagnosis is anterior chamber cleavage disorder (Jung wolff back stahl syndrome) which includes cerebellar hypoplasia, hypothyroidism, andtracheal stenosis^6^. Peters' anomaly with history of airway obstruction should always be screened fortracheal stenosis and hypothyroidism.

Preoperative echocardiography, neumimaging studies and other diagnostic studies should be done whenever indicated. The ability to detect associated systemic anomalies inthe early stage is important, because early treatment for those anomalies is essential for nonnal development. We have been referring such patients for examination to a paediatrician on their first visit. We feel that patients with Peters' anomaly be screened for systemic malformations and anaesthetic implication is based mainly on the presence of systemic anomalies and difficult airway.
